# Mitochondrial Localized STAT3 Is Involved in NGF Induced Neurite Outgrowth

**DOI:** 10.1371/journal.pone.0021680

**Published:** 2011-06-27

**Authors:** Lihan Zhou, Heng-Phon Too

**Affiliations:** 1 Department of Biochemistry, National University of Singapore, Singapore, Singapore; 2 Chemical Pharmaceutical Engineering, Singapore–Massachusetts Institute of Technology Alliance, Singapore, Singapore; University of Pittsburgh, United States of America

## Abstract

**Background:**

Signal transducer and activator of transcription 3 (STAT3) plays critical roles in neural development and is increasingly recognized as a major mediator of injury response in the nervous system. Cytokines and growth factors are known to phosphorylate STAT3 at tyrosine^705^ with or without the concomitant phosphorylation at serine^727^, resulting in the nuclear localization of STAT3 and subsequent transcriptional activation of genes. Recent evidence suggests that STAT3 may control cell function via alternative mechanisms independent of its transcriptional activity. Currently, the involvement of STAT3 mono-phosphorylated at residue serine^727^ (P-Ser-STAT3) in neurite outgrowth and the underlying mechanism is largely unknown.

**Principal Findings:**

In this study, we investigated the role of nerve growth factor (NGF) induced P-Ser-STAT3 in mediating neurite outgrowth. NGF induced the phosphorylation of residue serine^727^ but not tyrosine^705^ of STAT3 in PC12 and primary cortical neuronal cells. In PC12 cells, serine but not tyrosine dominant negative mutant of STAT3 was found to impair NGF induced neurite outgrowth. Unexpectedly, NGF induced P-Ser-STAT3 was localized to the mitochondria but not in the nucleus. Mitochondrial STAT3 was further found to be intimately involved in NGF induced neurite outgrowth and the production of reactive oxygen species (ROS).

**Conclusion:**

Taken together, the findings herein demonstrated a hitherto unrecognized novel transcription independent mechanism whereby the mitochondria localized P-Ser-STAT3 is involved in NGF induced neurite outgrowth.

## Introduction

The transcription regulator STAT3 mediates a myriad of biological functions from immune response to cell proliferation and differentiation [1], [2], [3]. It is critically involved in the development of the rodent central nervous system [4], [5], [6] and plays key roles in mediating the protective and regenerative effects of cytokines, growth factors, and hormones following injuries to brain, spinal cord and peripheral nerves [3]. An important and common feature of both neuronal differentiation and regeneration is the process of neurite outgrowth, where neurons sprout axons and dendrite to establish a network of connection. Adequate neurite initiation and outgrowth are essential pre-requisites to target innervation and formation of neuronal circuitry. This process is typically triggered by extracellular cues and regulated through a myriad of interactive signaling pathways. The involvement of STAT3 in the process and the underlying mechanisms are largely unexplored.

STAT3 can be activated by cytokines and growth factors such as interleukin 6 (IL6) [7], [8], epidermal growth factor (EGF) [9], [10], and brain-derived neurotrophic factor (BDNF) [11], by phosphorylating tyrosine at residue 705 (P-Tyr-STAT3). This results in the homo-dimerization and nuclear translocation of STAT3 and the subsequent activation of target gene transcription [12]. STAT3 can also be phosphorylated at serine^727^ by ligand activated MAPKs, PKC and other serine kinases [13]. Unlike tyrosine phosphorylation, the functional significance of serine phosphorylation of STAT3 remains controversial. Depending on the type of cells and the extracellular stimuli, serine phosphorylation could either enhance or suppress the transcriptional activity of tyrosine phosphorylated STAT3 [14], [15], [16]. Furthermore, STAT3 can be mono-phosphorylated at serine^727^ (P-Ser-STAT3) and induce target gene transcription [11]. Thus far, all the reported functions of STAT3 in neurite outgrowth are mediated by mono-phosphorylated P-Tyr-STAT3 or dual phosphorylated P-Tyr/Ser-STAT3 via transcriptional activation.

Recently, P-Ser-STAT3 was found to be localized in the mitochondria for the optimal function of the electron transport chain [17]. This mitochondrial function of STAT3 does not require the DNA binding domain or the tyrosine phosphorylation. P-Ser-STAT3 was also found to augment metabolic functions in mitochondria and mediate malignant transformation induced by oncogenic Ras [18]. As the development and maintenance of the nervous system requires balanced energy metabolism, it is tempting to speculate that the non-canonical functions of STAT3 in the mitochondria may contribute to neuronal differentiation.

NGF stimulation of the rat pheochromocytoma PC12 cells induces growth arrest and the elaboration of neurite outgrowth [19]. Multiple signaling pathways have been implicated in this process, acting through both transcription-dependent and -independent mechanisms. In PC12 cells, NGF has been reported to induce STAT3 serine but not tyrosine phosphorylation [11], [20], resulting in the induction of cyclin D1 expression and growth arrest [11]. However, with both reports, it is unknown if P-Ser-STAT3 is involved in NGF induced neurite outgrowth.

In this study, we tested the hypothesis that P-Ser-STAT3 mediates NGF induced neurite outgrowth via a transcription-independent mechanism. Serine but not tyrosine dominant negative mutant of STAT3 was found to impair NGF induced neurite outgrowth. Unlike the previous report [11], NGF induced P-Ser-STAT3 was not detected in the nucleus. Unexpectedly, P-Ser-STAT3 was found to be localized to the mitochondria upon NGF stimulation, demonstrating for the first time, a ligand induced phosphorylation on serine^727^ residue of STAT3 in mitochondria. Using mitochondria targeted STAT3 mutants, mitochondrial P-Ser-STAT3 was found to regulate NGF induced neurite outgrowth and the production of ROS. Taken together, these findings provided novel insights into an unconventional, transcription-independent mechanism whereby mitochondria localized STAT3 mediates NGF induced neurite outgrowth.

## Results

### NGF induced sustained STAT3 serine but not tyrosine phosphorylation in PC12 cells and cortical neuron

**Figure 1:**
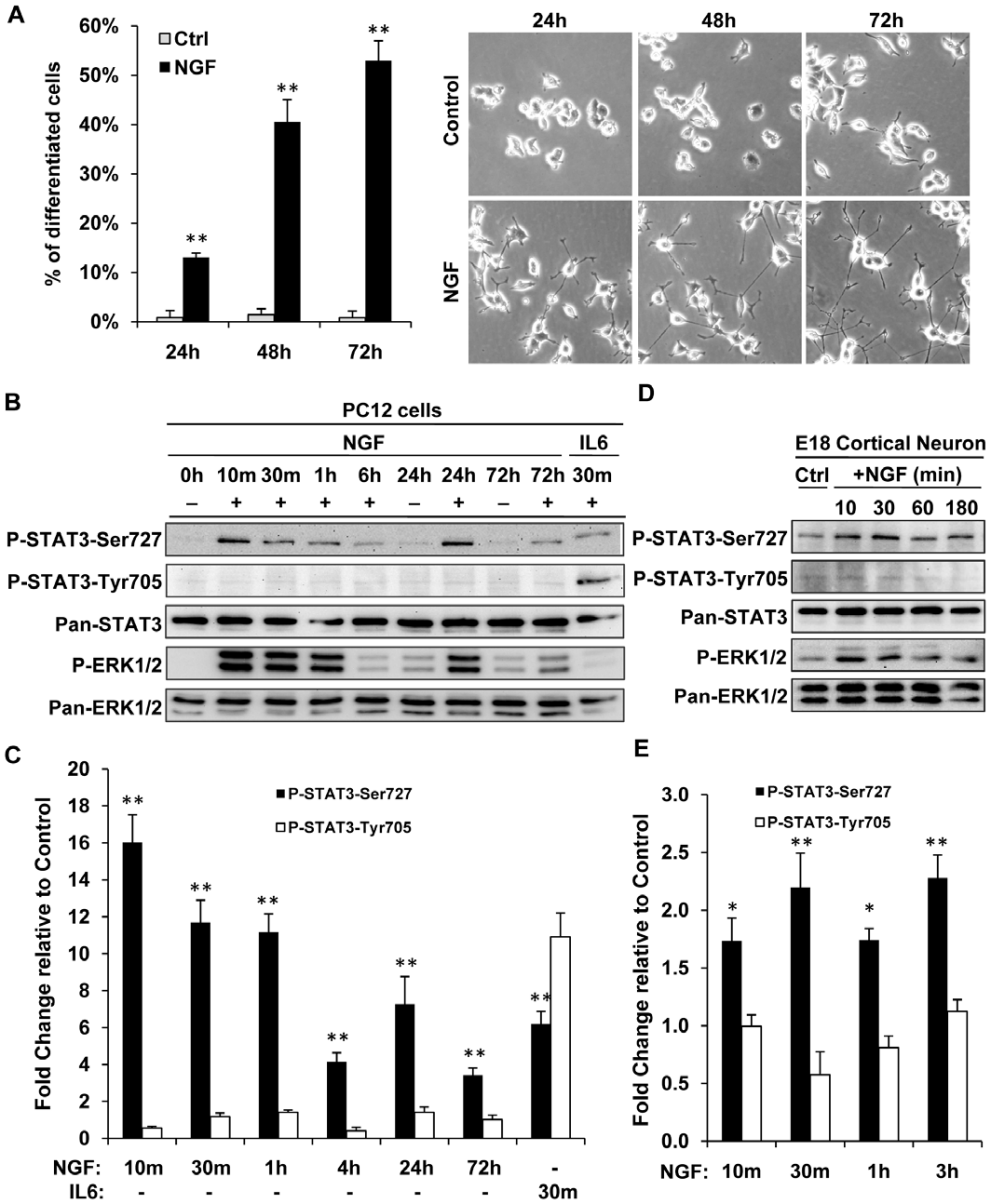
NGF induced sustained STAT3 serine^727^ but not tyrosine^705^ phosphorylation in PC12 and embryonic cortical neurons. PC12 cells were serum-deprived for 16 h and subsequently treated with NGF (50 ng/ml) for 72 h. *A.* NGF induced progressive neurite outgrowth in PC12 cells. Percentage of PC12 cells bearing neurite twice the body length (differentiated) was quantified after 24, 48 and 72 h of NGF treatment. Representative images of control and NGF treated cells are shown for each of the time point. *B.* Total cell lysates were collected over 72 h and analyzed by immunobloting and quantified (*C*). NGF induced sustained STAT3 serine but not tyrosine phosphorylation over 72 h, whereas IL6 induced both STAT3 serine and tyrosine phosphorylation. *D.* Rat embryonic cortical neurons were isolated and cultured in vitro for 72 h before NGF stimulation (100 ng/ml) for up to 3 h. Total cell lysates were analyzed and quantified (*E*). Significant differences in P-Ser or P-Tyr-STAT3 levels between ligand stimulated samples and respective controls were calculated using the paired Student's t-test. A value of p<0.05 was considered significant (**p<0.01; *p<0.05).

NGF induced progressive neurite outgrowth in PC12 cells (Fig. 1A). Within 72 h, greater than 50% of cells were found to bear neurite twice the cell body length. To gain insights into the involvement of STAT3 in this process, the phosphorylation state of STAT3 was investigated upon NGF stimulation. In agreement with previous reports [11], [20], STAT3 was rapidly (within 10 min) phosphorylated at serine^727^ upon NGF stimulation (Fig. 1B). The serine^727^ residue of STAT3 remained phosphorylated over a period of 72 h. No significant increase in STAT3 tyrosine^705^ phosphorylation was observed in NGF treated PC12 cells over the period of 72 h. A concomitant activation of ERK1/2 was also observed (lower panel), consistent with the previous reports [21], [22]. As a control, IL6 induced both tyrosine and serine phosphorylation of STAT3 in PC12 cells.

Neurotrophin receptors are expressed in cerebral cortical neuron *in vivo* [23] and *in vivo* [24]. In cortical neuron, BDNF induced a transient phosphorylation of STAT3 at tyrosine^705^ and a sustained phosphorylation at serine^727^ residue [11]. Whether NGF may similarly induce the phosphorylation of STAT3 at these sites is currently unknown. To address this possibility, primary cortical neurons were treated with NGF. Unlike BDNF [11], NGF induced a time dependent serine but not tyrosine phosphorylation of STAT3 in primary cortical neurons (Fig. 1C), extending the observations found in PC12.

### STAT3 serine DN mutant impaired NGF induced neurite outgrowth

**Figure 2:**
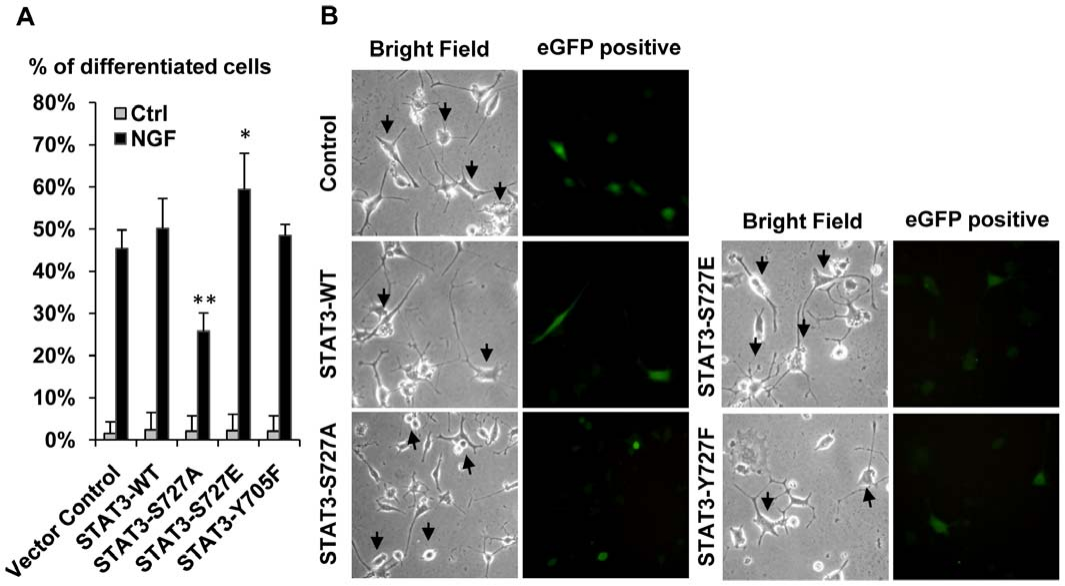
STAT3-Ser727Ala dominant negative mutant attenuated NGF induced neurite outgrowth in PC12 cells. PC12 cells were transiently infected with different STAT3 mutants using a retro-viral vector co-expressing eGFP (pQCXI-eGFP). Mutant expressing cells were identified by eGFP expression. Infected PC12 cells were serum deprived for 16 h and treated with 50 ng/ml NGF for 48 h. *A.* Neurite outgrowth from wild type and STAT3 mutants expressing PC12 cells was examined. Number of cells bearing at least one neurite twice the cell body length was scored. Significant differences between the percentages of neurite bearing cells in control and STAT3 mutant expressing cells were calculated using the paired Student's t-test. A value of p<0.05 was considered significant (**p<0.01; *p<0.05). *B.* Representative bright field and florescent images of control and STAT3 mutant expressing PC12 cells 48 h after NGF treatment. Arrows in bright field images point to STAT3 mutant expressing cells identified by eGFP co-expression.

To test the hypothesis that P-Ser-STAT3 is involved in NGF induced neurite outgrowth, wild type and mutant STAT3 were transiently expressed in PC12 cells. All STAT3 constructs co-expressed enhanced green florescence protein (eGFP) which served as a marker for gene transfer. STAT3 serine (S727A) but not tyrosine (Y705F) dominant negative mutant was found to attenuate NGF induced neurite outgrowth significantly (Fig. 2). In addition, expression of the STAT3 serine constitutive active mutant (S727E) resulted in a small but significant enhancement of NGF induced neurite outgrowth, lending further evidence that P-Ser-STAT3 was involved in neurite outgrowth.

### NGF induced P-Ser-STAT3 was undetectable in nucleus

**Figure 3:**
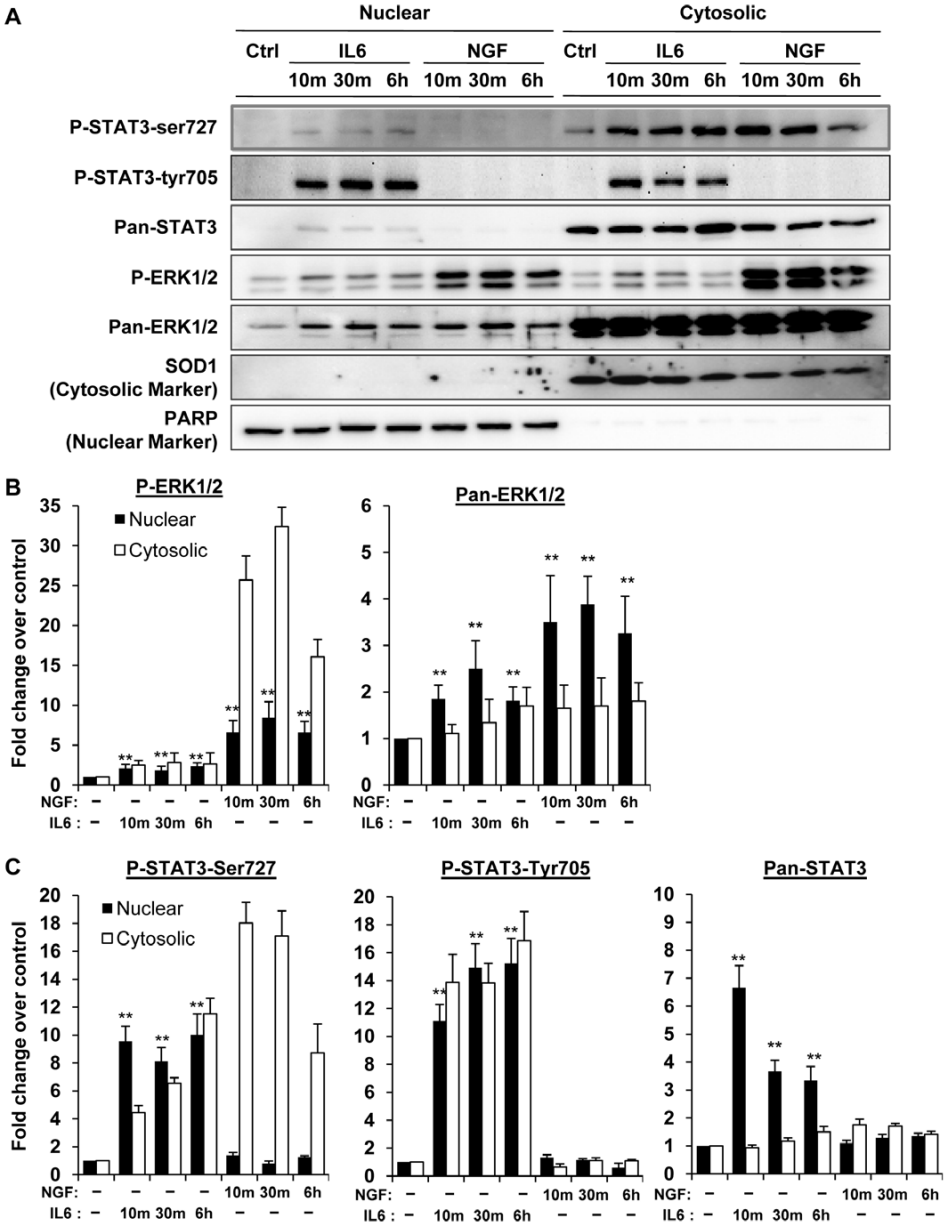
NGF did not induce STAT3 nuclear translocation. PC12 cells were serum-deprived for 16 h and subsequently treated with NGF (50 ng/ml) or IL6 (50 ng/ml) for 10 min, 30 min and 6 h respectively. Nuclear and cytosolic fractions were extracted and analyzed by immunobloting. The purity of the isolated nuclear and cytosolic fractions was verified by immunobloting analysis of nuclear marker PARP and cytosolic marker SOD1. Fold changes in nuclear and cytosolic Phospho & Pan-ERK (*B*) and Phospho and Pan-STAT3 (*C*) were quantified. Nuclear proteins were normalized by PARP whereas cytosolic proteins were normalized by SOD1. Significant differences in normalized nuclear levels of various proteins between control and ligand stimulated samples were calculated using the paired Student's t-test. A value of p<0.05 was considered significant (**p<0.01; *p<0.05).

Nuclear localization of STAT3 is known to be essential for the transcriptional activation of target genes. The temporal expressions of these target genes are likely to be influenced by the kinetics of STAT3 nuclear translocation and the duration of its presence in the nucleus. The previous report has briefly demonstrated the presence of P-Ser-STAT3 in nucleus [11] but it is not known if the nuclear localization of the phosphorylated STAT3 is temporally controlled. To address this, we investigated the time course of NGF induced P-Ser-STAT3 nuclear translocation. IL6, which is known to induce rapid nuclear translocation of STAT3 in PC12 cells [25], was used as a control. Nuclear and cytosolic fractions were prepared from PC12 cells stimulated with either IL6 or NGF and subsequently analyzed (Fig. 3A). Both IL6 and NGF induced rapid phosphorylation and increased nuclear localization of ERK (Fig. 3B). Upon IL6 stimulation, phosphorylated STAT3 was detected in both nuclear and cytosolic fractions within 10 min and was sustained over a period of 6 h (Fig. 3C). Contrary to the previous report [11], STAT3 was not detectable in the nucleus when the cells were stimulated with NGF over a period of 6 h (Fig. 3C). This observation indicated that serine phosphorylation alone did not result in detectable nuclear localization of STAT3, suggesting that P-Ser-STAT3 may be involved in NGF induced neurite outgrowth through a transcription independent mechanism.

### STAT3 was localized to mitochondria and was serine phosphorylated upon NGF stimulation

**Figure 4:**
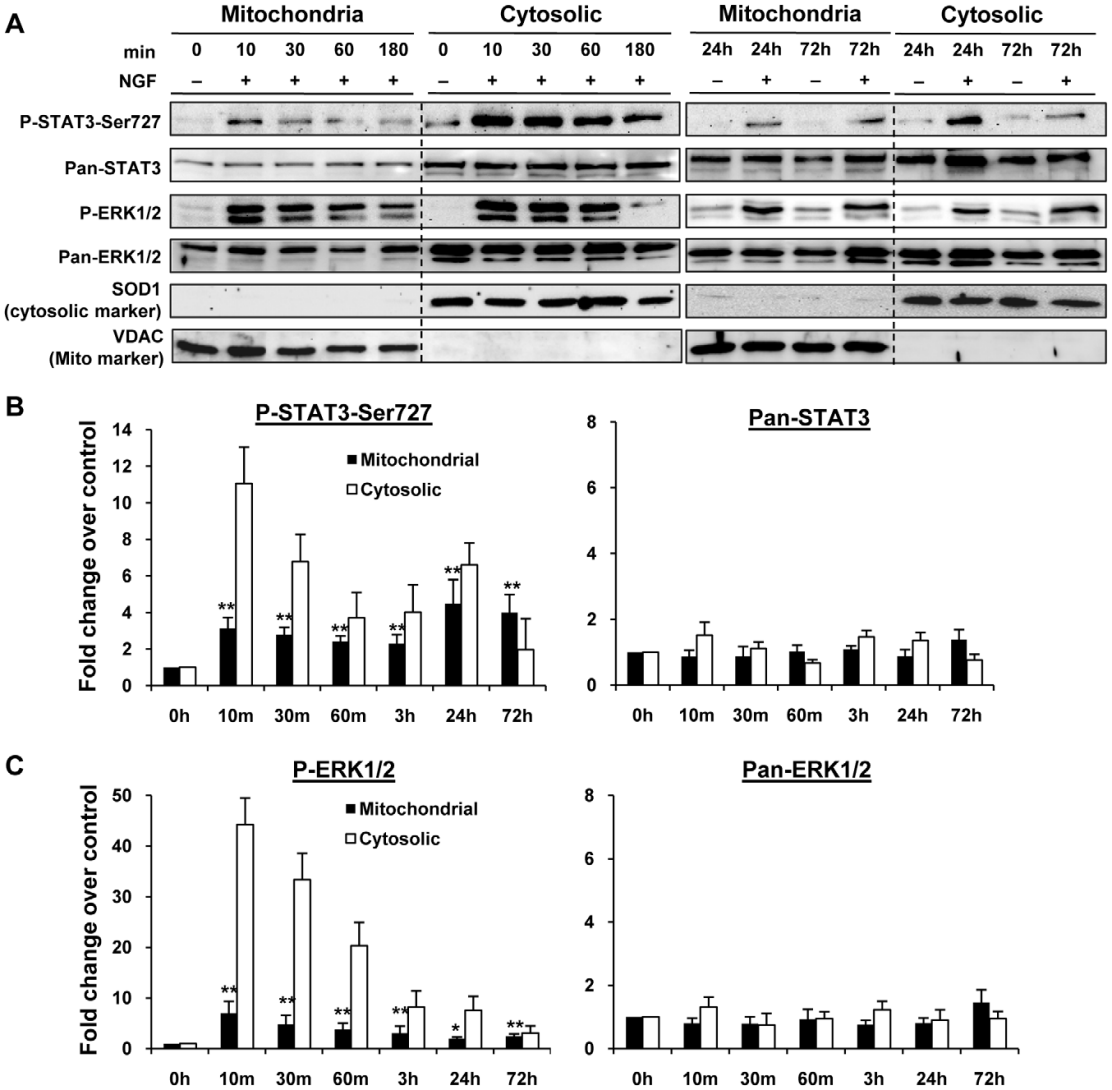
STAT3 was localized to mitochondria and was serine phosphorylated upon NGF stimulation. PC12 cells were serum-deprived for 16 h and subsequently treated with NGF (50 ng/ml) over 72 h. Mitochondrial and cytosolic fractions were extracted at different time points and analyzed by immunobloting. The purity of the isolated mitochondrial and cytosolic fractions was verified by immunobloting analysis of mitochondrial marker VDAC and cytosolic maker SOD1. Western blot images were shown in (*A*) and quantifications in (*B, C*). Mitochondrial proteins were normalized by VDAC whereas cytosolic proteins were normalized by SOD1. Significant differences in mitochondrial levels of various proteins between control and ligand stimulated samples were calculated using the paired Student's t-test. A value of p<0.05 was considered significant (**p<0.01; *p<0.05).

Recently, P-Ser-STAT3 was shown to be localized in the mitochondria of non-neuronal cells and regulated cellular functions independent of its transcriptional activities [17]. We next tested the hypothesis that NGF induced P-Ser-STAT3 may similarly be localized to the mitochondria. Mitochondrial and cytosolic fractions were isolated from NGF stimulated PC12 cells and total STAT3, as well as P-Ser-STAT3, were immunoblotted. Interestingly, P-Ser-STAT3 was detected in the mitochondria fraction when the cells were stimulated with NGF as early as 10 min (Fig. 4A, B) and the presence of P-Ser-STAT3 was detectable over a period of 72 h.

To verify that mitochondrial P-Ser-STAT3 signal was not due to a contamination from the cytoplasm, immunoblot intensity ratios of P-Ser-STAT3 to the cytosolic marker SOD1 in mitochondria and cytosol were compared as previously described [17]. While the intensity ratio of P-Ser-STAT3 to SOD1 in the cytosol was ∼1.2 at 10 min, the ratio of P-Ser-STAT3 to SOD1 in the mitochondria at 10 min was ∼8. If the detected P-Ser-STAT3 in the mitochondria was due to cytosolic contamination, the ratio of P-Ser-STAT3 to SOD1 would be ∼1.

Since STAT3 has been shown to shuttle between nucleus and cytosol, STAT3 may similarly shuttle between mitochondria and cytoplasm. Intriguingly, total mitochondrial STAT3 was not increased by NGF stimulation, suggesting that STAT3 may be constitutively present in mitochondria. This observation suggested an intriguing possibility that STAT3 may be directly phosphorylated in the mitochondria upon NGF stimulation. Consistent with this suggestion is that ERK, which has been shown to phosphorylate STAT3 at serine^727^, was robustly activated in the mitochondria upon NGF treatment (Fig. 4A, C). These observations supported the hypothesis that STAT3 may be phosphorylated in mitochondria by activated ERK or other kinases.

It is of interest to note that the total amount of STAT3 in the mitochondria of PC12 cells was about 13% of cytosolic STAT3, consistent with the earlier report where mitochondrial STAT3 amounted to one-tenth of that found in the cytosol in mouse liver and heart [17]. In addition, the amount of mitochondrial ERK in PC12 cells was about 25% of cytosolic ERK, similar to the previous report where the mitochondrial and cytosolic ERK account for 15% and 55% of total ERK in Hela cells, respectively [26]. The agreement between our data and previous reports further supported the reliability of our method and provided indication of a possible role of mitochondrial P-Ser-STAT3 downstream of NGF.

**Figure 5:**
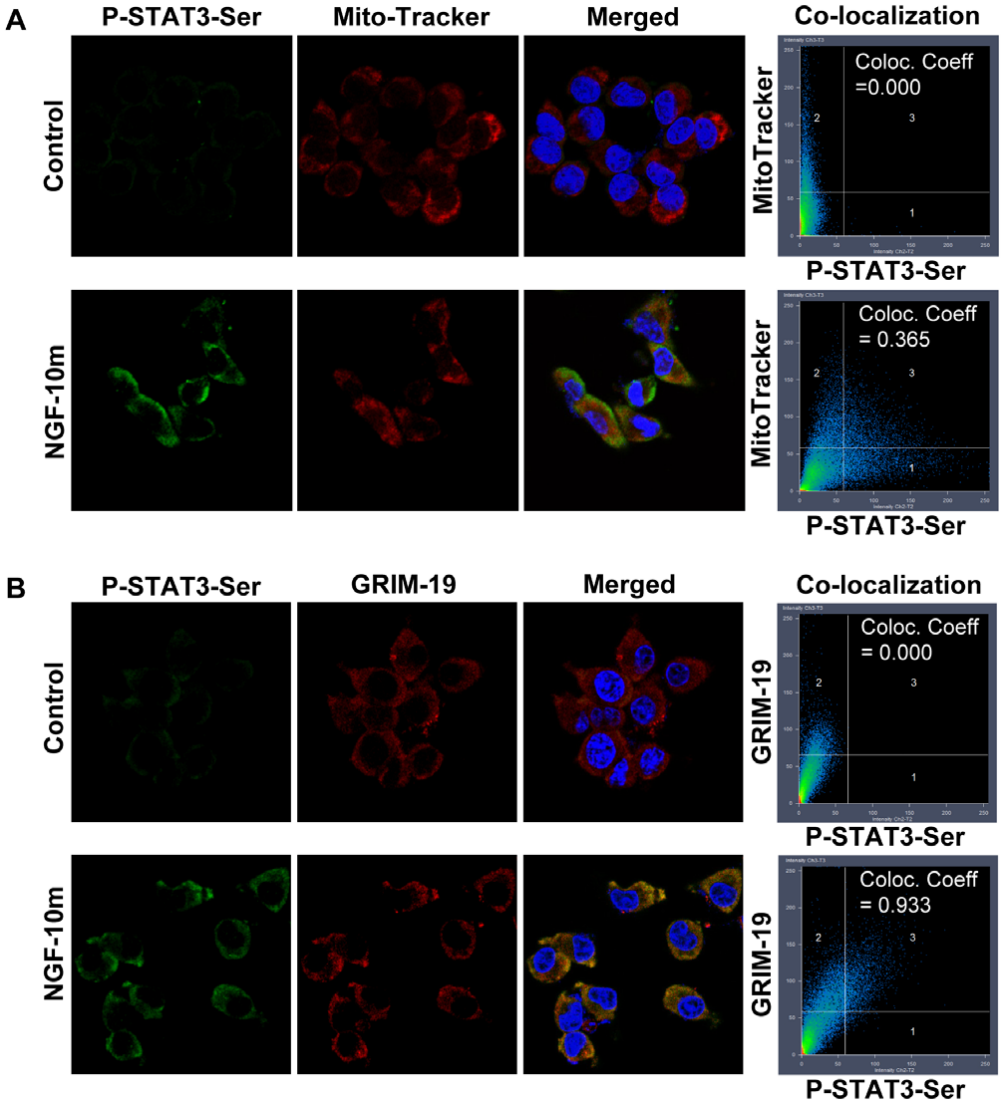
P-Ser-STAT3 was co-localized with MitoTracker and GRIM-19 in PC12 cells. NGF stimulated (10 min) PC12 cells were co-stained for P-Ser-STAT3 & MitoTracker (*A*) or P-Ser-STAT3 & GRIM-19 (*B*). Confocal images of control and NGF stimulated cells of the individual and merged channels are shown. Also shown here are the intensity correlations and co-localization coefficients between P-Ser-STAT3 and MitoTracker or GRIM-19.

**Figure 6:**
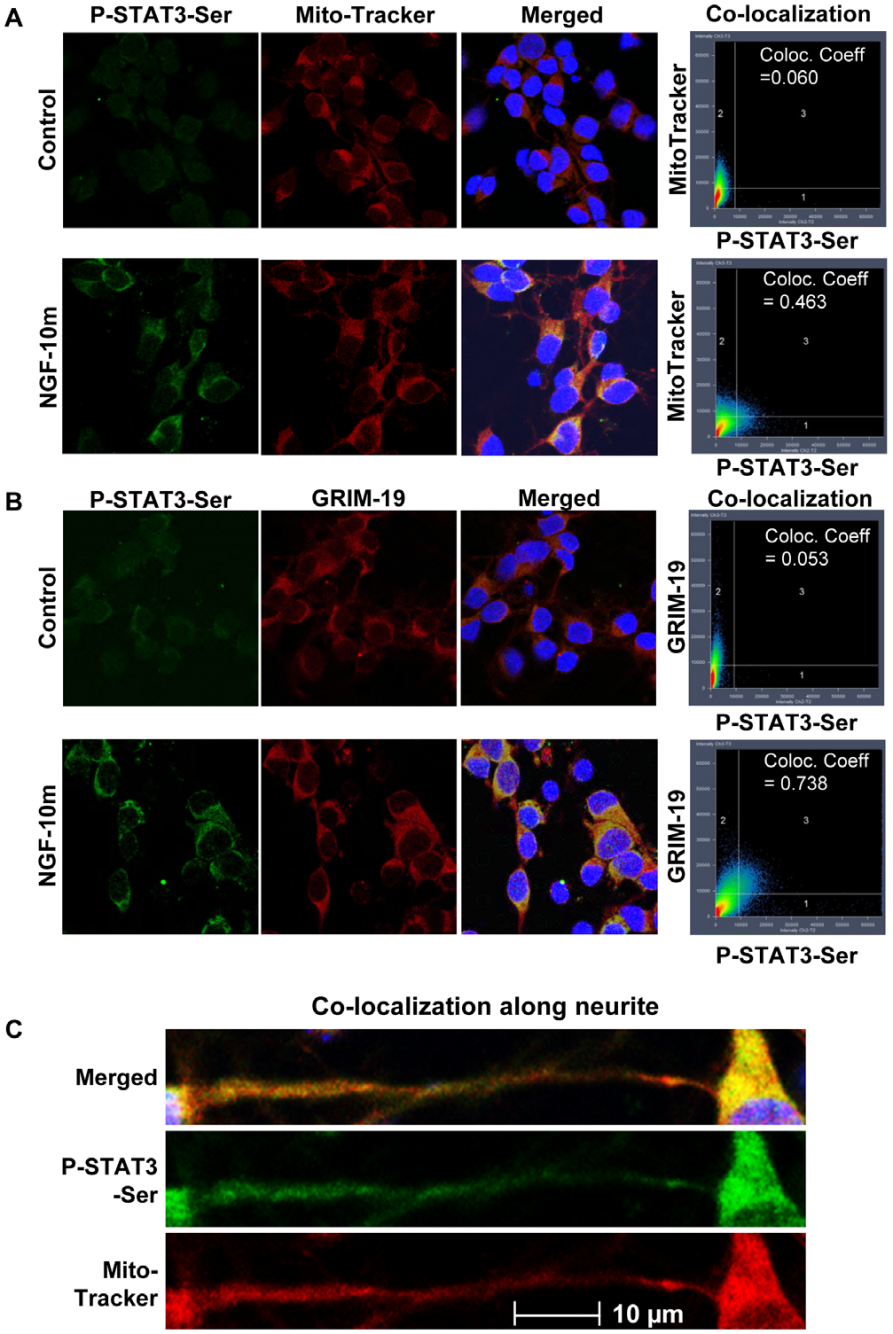
P-Ser-STAT3 was co-localized with MitoTracker and GRIM-19 in rat embryonic cortical neuron. NGF stimulated (10 min) cortical neurons were co-stained for P-Ser-STAT3 & MitoTracker (*A, C*) or P-Ser-STAT3 & GRIM-19 (*B*). Confocal images of control and NGF stimulated cells of the individual and merged channels are shown. Also shown here are the intensity correlations and co-localization coefficients between P-Ser-STAT3 and MitoTracker or GRIM-19.

Next, we verified the existence of P-Ser-STAT3 in the mitochondria immunocytochemically. NGF stimulation significantly increased the florescent intensity of P-Ser-STAT3 and the co-localization (*Co-localization Coefficient = 0.365*) with the specific mitochondria marker, Mito-Tracker (Fig. 5A). Furthermore, NGF induced P-Ser-STAT3 was also found to co-localize (*Co-localization Coefficient = 0.933*) with GRIM-19 (Fig. 5B), a known STAT3 binding partner and a component of the mitochondrial electron transport complex I [17], [27]. To test if the mitochondrial localization of STAT3 was restricted to the transformed cell line of PC12, we extended the study to rat embryonic cortical neurons. Consistent with the observations in PC12 cells, NGF induced P-Ser-STAT3 was also found to co-localize with MitoTrakcer (Fig. 6A, *Co-localization Coefficient = 0.463*) as well as GRIM-19 (Fig. 6B, *Co-localization Coefficient  = 0.738*) in primary cortical neurons. More intriguingly, P-Ser-STAT3 was found to co-localize with MitoTracker not only in the cell body but also along the neurites (Fig. 6C). Since local mitochondrial function is important for growth cone activity [28], axonal branching [29], and synapse formation [30], it is tempting to speculate that NGF may regulate local mitochondrial function and neurite outgrowth through mitochondrial localized P-Ser-STAT3. Taken together, our data from both subcellular fractionation and immunocytochemical staining experiments support the notion that P-Ser-STAT3 was localized to the mitochondria of neuronal cells upon NGF stimulation.

### STAT3 serine phosphorylation was temporally regulated by MAPKs and PKC

**Figure 7:**
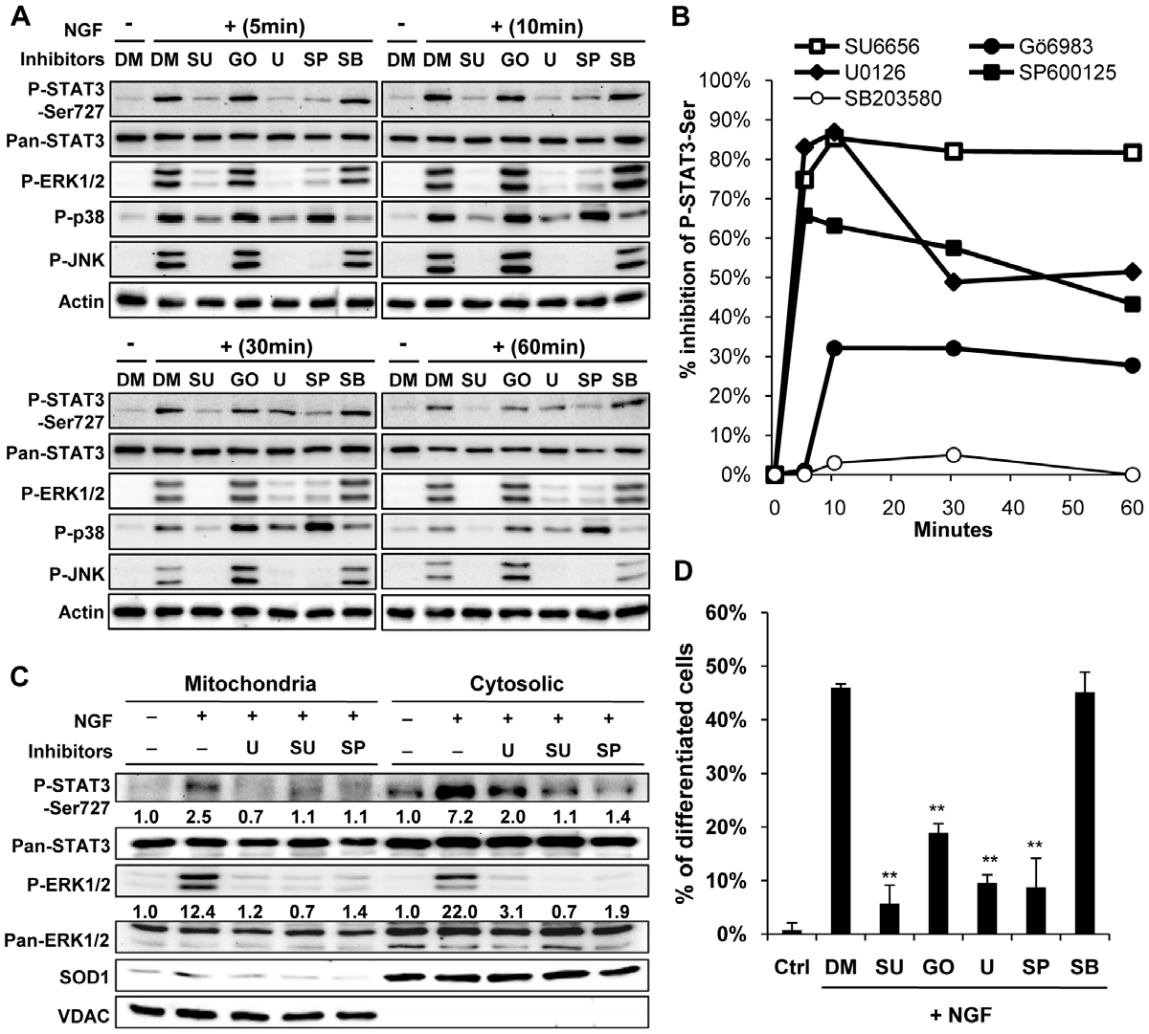
NGF induced STAT3 serine phosphorylation was temporally regulated by multiple kinases. PC12 cells were serum-deprived for 16 h and pre-treated with DMSO (DM, 0.1%, control), SU6656 (SU, 1 µM), Gö6983 (GO, 5 µM), U0126 (U, 10 µM), SP600125 (SP, 10 µM), and SB203580 (SB, 10 µM) for 1 h before stimulated with 50 ng/ml NGF in the presence of respective inhibitors. *A.* Total cell lysates were harvested from control and inhibitor treated cells after 5, 10, 30 or 60 min of NGF stimulation and analyzed by immunobloting. *B.* Percentage inhibition of NGF induced STAT3 serine phosphorylation by various signalling inhibitors, % inhibition  =  (P-Ser-STAT3_DMSO_ - P-Ser-STAT3_Inhibitor_)/P-Ser-STAT3_DMSO_ x 100%. Quantified P-Ser-STAT3 immunoblot intensity was normalized to respective Pan-STAT3 intensity. *C.* Mitochondrial and cytosolic fractions were extracted from NGF stimulated PC12 cells (10 min) pretreated with U0126, SU6656 or SP600125, and immunoblotted. The fold changes in normalized P-Ser-STAT3 and P-ERK were quantified and shown below the blot. *D.* The effect of various inhibitors on NGF induced neurite outgrowth was quantified in PC12 cells subjected to 48 h of NGF stimulation in the presence of the inhibitors. Significant differences between the percentages of differentiated cells in control and inhibitor treated PC12 cells were calculated using the paired Student's t-test. A value of p<0.05 was considered significant (**p<0.01; *p<0.05).

Previous report showed that inhibition of MEK-ERK pathway alone was insufficient to abolish NGF induced P-Ser-STAT3 in PC12 cells [11], suggesting the requirement of additional pathways for maximal STAT3 activation. To assess the possible involvement of various MAPK pathways (ERK, JNK and p38) and PKC in NGF induced STAT3 serine phosphorylation, PC12 cells were pretreated with selective pharmacological inhibitors, and subsequently stimulated with NGF for varying periods of time. Consistent with previous reports [31], [32], Src was found to be a crucial signaling intermediate mediating NGF activation of various MAPK pathways. Pre-treatment of PC12 cells with SU6656 substantially reduced NGF induced phosphorylation of ERK, JNK and p38. Similarly, SU6656 was able to abolish NGF induced STAT3 serine phosphorylation (Fig. 7). Another Src inhibitor PP2 likewise abolished NGF induced P-Ser-STAT3 (data not shown). Among the three MAPKs investigated, inhibition of ERK and JNK but not p38 activation was found to attenuate STAT3 serine phosphorylation (Fig. 7B). Phosphorylation of STAT3 serine^727^ was also inhibited by the broad spectrum protein kinase C inhibitor, Gö6983 (Fig. 7B). Taken together, the data suggested that NGF induced P-Ser-STAT3 was temporally regulated by multiple signaling pathways including ERK, JNK and PKC. To test the possibility that ERK and JNK are involved in serine phosphorylation of mitochondrial STAT3, mitochondrial fraction was isolated from PC12 cells pre-treated with SU6656, U0126 and SP600125. All three inhibitors were found to attenuate the phosphorylation of cytosolic and mitochondria STAT3 (Fig. 7C), indicating that pathways involving ERK, JNK and Src were indeed involved in NGF induced mitochondrial P-Ser-STAT3.

Intriguingly, inhibition of Src, ERK, JNK and PKC but not p38 pathway was found to attenuate NGF induced neurite outgrowth (Fig. 7D). The correlation between the activations of these pathways in NGF induced P-Ser-STAT3 and neurite outgrowth was suggestive of STAT3 acting as a downstream effector that contributes to neurite outgrowth.

### Mitochondrial STAT3 is an important mediator of NGF induced neurite outgrowth

**Figure 8:**
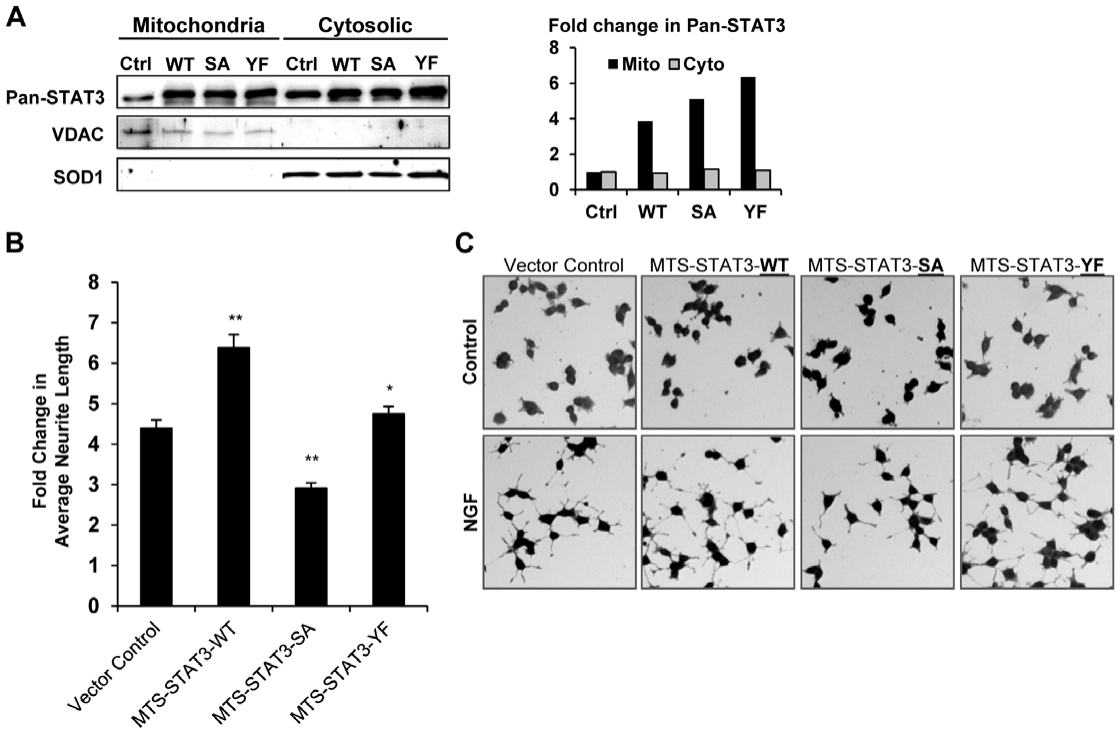
Mitochondrial STAT3 was involved in NGF induced neurite outgrowth. PC12 clones stably expressing mitochondria targeting wild type and mutant STAT3 were generated. *A.* Over-expression of STAT3 in mitochondria was verified by analyzing the level of total STAT3 in mitochondria of control and infected cells. Mitochondrial STAT3 were normalized by VDAC and cytosolic STAT3 normalized by SOD1. Fold changes in mitochondrial and cytosolic STAT3 level in infected cells over control were calculated. *B.* Control and MTS-STAT3 expressing PC12 clones were serum deprived and stimulated with 50 ng/ml NGF for 48 h. The average neurite length per cell was quantified using HCA-Vision. Fold changes in average neurite length in NGF treated cells over control were calculated for vector control PC12 cells and each of the MTS-STAT3 mutant expressing cells. Significant differences between control and mutant expressing PC12 cells were calculated using the paired Student's t-test. A value of p<0.05 was considered significant (**p<0.01; *p<0.05). Representative images of control and NGF treated cells are shown in *C.*

To investigate the role of mitochondrial P-Ser-STAT3 in NGF induced neurite outgrowth, mitochondria targeting wild type and mutant STAT3 (MTS-STAT3) were constructed by fusing the mitochondrial targeting sequence of cytochrome c oxidase subunit VIII to the N terminus of STAT3. This sequence has been reported to target the protein of interest to the inner mitochondrial membrane [33], where mitochondrial STAT3 was thought to exert its function. Such MTS-STAT3 constructs have been used successfully to investigate the functions of mitochondrial STAT3 in respiration [17] and oncogenic transformation [18]. Subcellular fractionation of PC12 cells stably expressing wild type and mutant MTS-STAT3 showed an over-expression of STAT3 in the mitochondrial (4-6 fold) but not in the cytosolic fraction (Fig. 8A). NGF induced neurite outgrowth was then studied using these constructs. Remarkably, mitochondria targeted serine dominant negative mutant of STAT3 (MTS-STAT3-SA) attenuated NGF induced neurite outgrowth (Fig. 8B, C). In contrast, wild type (MTS-STAT3-WT) and tyrosine dominant negative mutant (MTS-STAT3-YF) of STAT3 were found to enhance NGF induced neurite outgrowth. These results demonstrated the involvement of mitochondrial P-Ser-STAT3 in NGF induced neurite outgrowth.

### NGF stimulated ROS production and the involvement of mitochondrial STAT3

**Figure 9:**
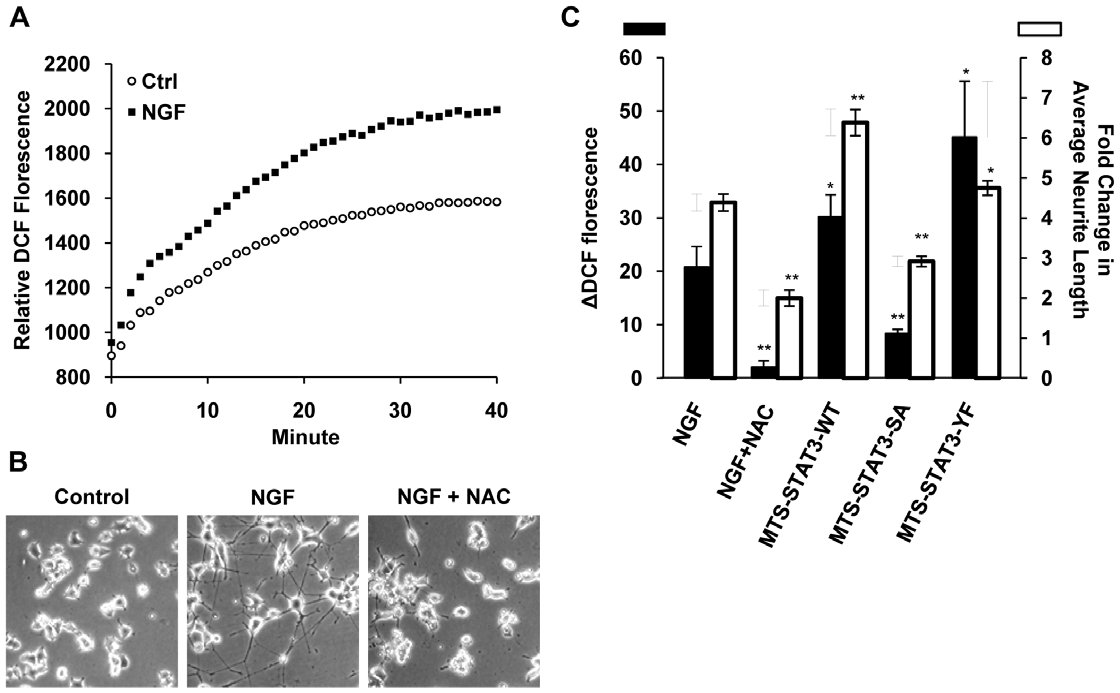
NGF induced ROS was partly mediated by mitochondrial STAT3. *A.* To quantify NGF stimulated production of ROS, PC12 cells were preloaded with DCFHDA (10 µM) for 10 min and stimulated with NGF (50 ng/ml). The florescent intensity of the oxidized product DCF was measured over the next 40 min. *B.* Representative images of control and NGF treated PC12 cells with and without NAC pretreatment (10 mM) are shown in. *C*. The rate of ROS production in NGF treated PC12 cells was calculated using the following formula, ΔDCF/min = (ΔDCF_NGF,0–30_ _min_ – ΔDCF_Ctrl,0–30_ _min_)/30. The rate of ROS production in PC12 cells stably expressing various MTS-STAT3 mutants was compared with that in non-simulated and NAC pre-treated vector control PC12 cells. Significant differences between the rates of ROS production were calculated using the paired Student's t-test. A value of p<0.05 was considered significant (**p<0.01; *p<0.05). The effect of MTS-STAT3 mutants on rate of ROS production (black bar, primary vertical axis) was correlated with the effect on NGF induced neurite outgrowth (white bar, secondary vertical axis).

NGF stimulation has previously been shown to elevate ROS level in PC12 cells, contributing to NGF induced neurite outgrowth [34]. Recently, it was suggested that NGF may modulate ROS level in PC12 cells by regulating mitochondrial functions [35]. Since mitochondrial P-Ser-STAT3 was reported to modulate mitochondrial electron transport activities and the rate of oxygen consumption in non-neuronal cells [17], we tested the hypothesis that P-Ser-STAT3 may be involved in NGF regulation of ROS level in PC12 cells. Consistent with the previous report [34], an increase in total intracellular ROS was observed in PC12 cells when stimulated by NGF (Fig. 9A). As expected, pre-incubation of PC12 cells with the antioxidant N-acetylcysteine (NAC) significantly blocked NGF induced ROS production (Fig. 9C) and attenuated NGF induced neurite outgrowth (Fig. 9B).

NGF induced ROS production was then compared in wild type and cells stably expressing mitochondria targeted STAT3 mutants. Interestingly, we observed a significant reduction in NGF induced ROS production in cells expressing MTS-STAT3-SA mutant, and an increase in cells expressing MTS-STAT3-WT and YF mutants (Fig. 9C, black bar). The effects of STAT3 mutants on NGF induced ROS correlated well with their effects on NGF induced neurite outgrowth (Fig. 9C, white bar), indicative of a role of mitochondrial P-Ser-STAT3 in the regulation of NGF induced ROS production and neurite outgrowth.

## Discussion

Originally identified as a key mediator of cytokine induced inflammation and immunity, STAT3 has now been shown to regulate a myriad of other biological processes. STAT3 is known to be activated by a variety of ligands involved in neurite outgrowth [7], [8], [11], [36], [37], [38], [39]. To date, all reports supporting the involvement of STAT3 in neurite outgrowth are based on observations of the effects of either P-Tyr-STAT3 or P-Tyr/Ser-STAT3 [7], [8], [11]. Furthermore, there is no evidence as yet that mitochondrial localized P-Ser-STAT3 is involved in neurite outgrowth. The recent reports of NGF activated P-Ser-STAT3 in PC12 cells, did not demonstrate the contribution of phosphorylated serine^727^ STAT3 to neurite outgrowth [11], [20]. This is rather surprising, as PC12 cells are commonly used as a model for neuritogenesis induced by a variety of agents, including NGF. To the best of our knowledge, this is the first report that demonstrated the involvement of P-Ser-STAT3 in neurite outgrowth and the unexpected phosphorylation on serine^727^ residue of STAT3 in mitochondria, induced by the neurotrophic factor NGF.

It is known that P-Tyr-STAT3 and P-Tyr/Ser-STAT3 translocate to the nucleus and mediate neurite outgrowth via transcriptional activation [7], [8]. In this study, IL6 induced the efficient nuclear localization of P-Tyr/Ser-STAT3 whereas NGF induced P-Ser-STAT3 was undetectable in the nucleus even after 6 h of stimulation, an observation in contrast to the previous report [11]. Instead, NGF was found to increase the level of P-Ser-STAT3 in mitochondria with no observable change in the total level of mitochondrial STAT3, suggesting the possibility that existing mitochondrial STAT3 may be phosphorylated directly by kinases, e.g. ERK 1/2. Such hypothesis is supported by our observation that ERK1/2 is robustly activated in mitochondria upon NGF stimulation. This is consistent with previous findings that ERK1/2 can be activated in mitochondria [40], [41], which in turn phosphorylated other mitochondrial proteins such as steroidogenic acute regulatory protein [40]. However, our data does not rule out the possibility that cytosolic P-Ser-STAT3 may be exchanged with non-phosphorylated mitochondrial STAT3 through a bi-directional trafficking mechanism. Yet it remains unclear how STAT3, which lack a putative mitochondria targeting sequence, can be translocated into the mitochondria. Binding to and translocation along with mitochondria targeting partner/s may yet be another possibility for P-Ser-STAT3 to be preferentially localized to the mitochondria. We did notice that P-Ser-STAT3 was highly co-localized with GRIM-19, a cytosolic translated component of mitochondrial complex I. Whether STAT3–GRIM-19 complex formed in the cytosol enabled STAT3 to be translocated to the mitochondria is currently under investigation.

It is increasingly recognized that mitochondria is not merely a cellular powerhouse, but a signaling hub where the bi-directional communication with the cytosolic components play an integral role in many physiological processes including neuronal survival [42], [43], [44]. The recent evidence of mitochondria localized P-Ser-STAT3 enhancing functions of the electron transport chain[17], the augmentation of oxidative phosphorylation and oxygen consumption [18], raised the interesting possibility that P-Ser-STAT3 may play a role in neurite outgrowth in PC12 by modifying mitochondria functions. Consistent with this hypothesis is that the over-expression of mitochondria targeted STAT3 serine dominant negative mutant (MTS-STAT3-SA) was found to attenuate NGF induced neurite outgrowth and the generation of ROS, products of the electron transport chain in mitochondria. The involvement of ROS in neurite outgrowths have been explored in both primary neurons [45] and cell lines [34], [35], [46], [47], [48], [49]. Several of these studies have shown that NGF induced neurite outgrowth in PC12 cells involves elevated ROS level [34], [35], [48], which may be modulated through the regulation of mitochondrial functions [35]. In addition, ROS was recently shown to regulate F-actin dynamics in Aplysia bag cell neuron growth cone, lending further evidence that ROS participates in neuritogenesis [45]. Similar to these reports [34], [45], [46], [47], [49], ROS in our study was quantified by a broad ROS sensor DCFHDA that does not discriminate different species of ROS. To unravel the precise biochemical mechanism of ROS in the regulation of neurite outgrowth, it may be necessary to take into account the different species of ROS. Whether the effect of mitochondrial P-Ser-STAT3 on ROS production directly impacts its involvement in neurite outgrowth is currently under investigation. Furthermore, it has not escaped our attention that the cytosolic P-Ser-STAT3 may also be involved in neurite outgrowth via mechanisms yet to be characterized. Nonetheless, our data demonstrated the novel and intriguing role of mitochondrial P-Ser-STAT3 in neurite outgrowth.

Depending on cell type and stimuli, multiple pathways have been implicated in the phosphorylation of serine residue of STAT3 [13], [50], [51]. The observation that NGF induced serine phosphorylation of STAT3 in PC12 is temporally regulated by ERK, JNK and PKC pathways is novel and intriguing. The kinetics of NGF activations of these pathways is indicative of a complex network of signaling integrations and feedbacks, consistent with earlier report that NGF induced ERK and JNK pathways interact closely [31]. In addition, NGF activation of ERK was shown to involve PKCε, a specific isoform of the PKC family [52]. Further studies to elucidate the involvement of different PKC isoforms in STAT3 serine phosphorylation as well as their interactions with the MAPK pathways may help to delineate the biochemical mechanisms underlying the temporal regulation of NGF induced P-Ser-STAT3.

In conclusion, this study demonstrated that P-Ser-STAT3 is intimately involved in NGF induced neurite outgrowth in PC12 cells. This function is dependent on the spatial cellular organization of P-Ser-STAT3 to the mitochondria and is correlated to the regulation of ROS production. Distinct signaling mechanisms involving MAPK and PKC contribute to the phosphorylation serine^727^ residue of STAT3. Taken together, this study has provided novel insights into an unconventional, transcription-independent mechanism whereby mitochondria localized STAT3 is involved in NGF induced neurite outgrowth.

## Materials and Methods

### Cell Culture

The rat pheochromocytoma cell line PC12 (American Type Culture Collection; catalog # CRL-1721) cells were grown in DMEM (Sigma, St. Louis, MO) supplemented with 5% Horse Serum (HS, Hyclone, Logan, UT), 10% heat-inactivated fetal bovine serum (FBS; Sigma), 100 units/ml penicillin (Pan Biotech, Germany) and 100 µg/ml streptomycin (Pan Biotech), at 37°C in a humidified atmosphere with 5% CO_2_. For studies of ligand induced signaling activation and neurite outgrowth, PC12 cells were serum starved in DMEM with 0.25% HS, 0.5% FBS and Penicillin/Streptomycin for 12–16 h before stimulated with 50 ng/ml of NGF (PreproTech, London, UK) or IL6 (PreproTech) in the same media. For inhibitor studies, PC12 cells were pre-treated with SU6656 (Sigma), PP2 (Tocris, Bristol, UK), U0126 (Promega), SP600125 (Tocris), SB203580 (Tocris), or Gö6983 (Sigma) for 1 h before stimulated with NGF in the presence of these inhibitors. Rat primary cortical neurons were isolated from embryonic day 18 (E18) rat embryos. All procedures were performed according to the guidelines by the Institutional Animal Care & Use Committee (IACUC) of NUS. This study was approved by NUS IACUC under a broader project titled “*Mechanism elucidation and application of global Transcriptional Machinery Engineering (gTME) to the modulation of the isoprenoid pathway for biosynthesis of pharmaceutical*” and given the animal work permits (072/09). Cortices were dissected and dissociated in Hank's balanced salt solution (Sigma), and plated on poly-D-lysine (Sigma) coated culture plates in Minimal essential medium (MEM, Sigma) supplemented with glucose (0.6% wt/vol, Sigma) and 10% FBS. After 24 h, MEM was replaced by Neurobasal medium (Invitrogen, Carlsbad, CA) containing B27 supplement (Invitrogen), 2 mM glutamine (Invitrogen), 100 units/ml penicillin and 100 µg/ml streptomycin and continued to culture for 48 h at 37°C in a humidified atmosphere with 5% CO_2_. On DIV 3 (3 days in vitro), primary cortical neurons were washed once with Neurobasal medium without B27 and kept in the same medium for 3 h before subjected to 100 ng/ml NGF stimulation.

### Construction of retroviral vectors containing wild type and mutant STAT3

Modified pXJ-40 vectors containing cDNAs for wild type and three mutant forms of STAT3a (Ser727Ala, Ser727Glu, Tyr705Phe) were kindly provided by Dr Cao Xinming (Institute of Molecular and Cellular Biology, Singapore). These wild type and mutant STAT3a constructs were sub-cloned into a modified murine retroviral pQCXI vector (Clontech, Mountain View, CA) that harbors an eGFP-coding sequence under the control of an internal ribosomal entry site (pQCXI-eGFP). To create mitochondria targeted STAT3 constructs, the mitochondrial targeting sequence (MTS, from cytochrome c oxidase subunit VIII) was first cloned by polymerase chain reaction (PCR). The MTS sequence was fused to the 5′ of wild type and mutant STAT3a by assembly PCR and subcloned into the retroviral pQCXI-P vector containing puromycin resistance gene as selection marker.

### Infection and Establishment of PC12 clones stably expressing MTS-STAT3 mutant

For transient expression experiment, PC12 cells were infected with wild type or mutant STAT3 in pQCXI-eGFP vector by retro-viral infection. To establish PC12 clones stably expressing mitochondria targeted STAT3, cells were infected with MTS-STAT3 in pQCXI-P vector by retro-viral infection and selected with 2 µg/ml Puromycin (Sigma), over a period of 2 months.

### Western blot analysis

PC12 cells and rat primary cortical neurons were washed once with PBS and subsequently lysed in lysis buffer containing 2% (w/v) SDS. Protein concentrations were quantified using the microBCA assay (Pierce, Rockford, IL). The protein samples were then separated by SDS-PAGE gel and probed with antibodies against Phospho-STAT3-ser727 (#9134, Cell Signalling Technology [CST], Beverly, MA), Phospho-STAT3-tyr705 (sc-8059, Santa Cruz), Pan-STAT3 (#9132, CST), Phospho-ERK1/2 (#9101, CST), Pan-ERK1/2 (#9102, CST), PARP (#9542, CST), VDAC (#4866, CST) or SOD1 (sc-11407, Santa Cruz). The protein bands were developed with Immobilon Western Chemilum HRP Substrate (Millipore, Billerica, MA) on ChemiDoc XRS system (Biorad). The band intensities were quantified using Quantity One 1-D Analysis software v4 (Biorad).

### Preparation of mitochondrial and cytosolic extracts

Mitochondrial and cytosolic extracts were prepared from PC12 cells using differential centrifugation, as previously described [17], [26]. Briefly, PC12 cells were washed twice with ice-cold phosphate buffer saline (PBS) and harvested by gentle scraping. Cells were pelleted by centrifugation at 500 x g for 5 min and resuspended in ice cold mitochondria extraction buffer (50 mM HEPES pH 7.4, 68 mM sucrose, 200 mM D-mannitol, 50 mM KCl, 2 mM MgCl_2_, 5 mM EDTA, 10 µg/ml aprotinin, 2 µg/ml pepstatin A, 10 µg/ml leupeptin, 50 mM NaF, 0.5 mM sodium vanadate, 20 mM glycerol phosphate) and incubated on ice for 15 min to allow swelling. The cells were dounce homogenized with 60 strokes. The unbroken cells and nuclei were removed by centrifugation at 800 x g for 10 min at 4°C. The supernatants were further centrifuged at 10,000 x g for 30 min at 4°C to obtain the crude mitochondrial fraction. The resulting supernatants were collected as the cytosolic extracts and the crude mitochondrial pellet was washed once with extraction buffer and collected by centrifugation at 10,000 x g for 30 min at 4°C.

### Preparation of nuclear and cytosolic extracts

The nuclear and cytosolic extracts were prepared using NE-PER® Nuclear and Cytoplasmic Extraction Reagents (Pierce), according to the manufacturer's instruction. Briefly, PC12 cells were washed twice with ice-cold PBS and harvested by gentle scraping. Cells were pelleted by centrifugation at 500 x g for 5 min and resuspended in ice cold CER I and incubated on ice for 10 min. The cells were lysed by addition of ice cold CER II and centrifuged at 16,000 x g for 5 min to separate the cytosolic fraction (supernatant) from the nucleus (pellet). The pellet was resuspended in ice cold NER and incubated on ice for 40 min with occasional vortexing (15 s after every 10 min of incubation). The nuclear extracts (supernatant) were separated from the debris by centrifugation at 16,000 g for 10 min.

### Differentiation and assessment of neurite outgrowth

Wild type or infected PC12 cells were seeded on Poly-D-Lysine coated 6-well cell culture plates (NUNC, Finland) overnight in DMEM supplemented with 5% HS and 10% FBS, and serum starved for 12–16 h. PC12 cells were then treated with 50 ng/ml NGF for 48 h to induce neurite outgrowth. For transient STAT3 mutant expression experiments, infected cells were identified by eGFP expression and those eGFP positive cells bearing at least one neurite with the length equivalent to two cell-body length were scored. More than 60 eGFP positive cells from three biological replicates were counted. NGF induced neurite outgrowth from PC12 clones stably expressing mitochondria targeted STAT3 mutants were analyzed using HCA-Vision (CSIRO, AU). Briefly, NGF treated PC12 cells were fixed with 4% paraformaldehyde (PFA; BDH Laboratory, UK). The cell bodies and neurites were stained with Imperial Stain (Pierce) and the nuclei with 1 µg/ml Hoescht 33342 (Sigma). All images of control and NGF treated wild type and mutant STAT3 expressing cells were acquired with identical imaging parameters using a Zeiss Axio Observer Z1 Inverted Microscope (Carl Zeiss, Germany). All images were batch analyzed using HCA-Vision through a 3-step analysis including Neuron Body Detection, Neurite Detection and Neurite Analysis, with identical parameters. Significant differences in neurite outgrowth between wild type and STAT3 mutant expressing PC12 cells were calculated using the paired Student's t-test. A value of p<0.05 was considered significant (**p<0.01; *p<0.05).

### Measurement of intracellular reactive oxygen species

PC12 cells were seeded overnight in Poly-D-Lysine coated 96-well plate (NUNC) and serum-starved in DMEM lacking phenol red (Sigma) supplemented with 0.25% HS and 0.5% FBS for 12–16 h. Prior to NGF stimulation, cells were loaded with 5 µg/ml 2′,7′-dichlorofluorescein diacetate (DCFHDA, Sigma) for 10 min at 37°C in the dark and washed once with DMEM lacking phenol red. The DCF fluorescence intensity upon NGF stimulation was measured by a SpectraMax GerminiXS spectrometer (Molecular Device, Sunnyvale, CA) with an excitation wavelength of 485 nm and emission wavelength of 525 nm, over the period of 40 min.

### Immunocytochemistry

Control and NGF treated PC12 Cells and rat embryonic cortical neurons were fixed with 4% paraformaldehyde in 1xPBS for 15 min at 37°C, subsequently permeabilized in 0.5% Triton-X100/PBS and blocked with normal goat serum (1∶10; Dako, Glostrup, Denmark) in 0.5% Triton X-100/PBS for 45 min at 37°C. The cells were then incubated with primary antibodies against Phospho-STAT3-ser727 (CST #9134, 1∶100 dilution) or GRIM-19 (Invitrogen #438900, 1∶100 dilution) in 0.3% Triton X-100/1% BSA/1xPBS overnight at 4°C and washed three times in PBS. Subsequently, the cells were incubated with goat anti-rabbit or goat anti-mouse fluorescent secondary antibody (Alexa Fluor 488/596; Invitrogen, CA) diluted 1∶400 in 0.3% Triton X-100/1% BSA/PBS for 2 h at 37°C. The cells were washed three times in 1xPBS and mounted. For Mitotracker labeling, live PC12 cells were pre-incubated with 200 nM MitoTracker® Red CMXRos (Invitrogen M7512) for 10 min before NGF stimulation. Image acquisition was performed using the Zeiss LSM710 with Axio Observer.Z1 confocal microscope equipped with fluorescence detection (Oberkochen, Germany). Images of control and NGF stimulated samples were taken with identical laser and optical settings. Colocalization coefficients between P-Ser-STAT3 and MitoTracker or P-Ser-STAT3 and GRIM-19 were analyzed with Zeiss ZEN software (v2010).

## References

[1] Devarajan E, Huang S (2009). STAT3 as a central regulator of tumor metastases.. Curr Mol Med.

[2] Yu H, Pardoll D, Jove R (2009). STATs in cancer inflammation and immunity: a leading role for STAT3.. Nature Reviews Cancer.

[3] Dziennis S, Alkayed NJ (2008). Role of signal transducer and activator of transcription 3 in neuronal survival and regeneration.. Rev Neurosci.

[4] Cattaneo E, Conti L, De-Fraja C (1999). Signalling through the JAK-STAT pathway in the developing brain.. Trends Neurosci.

[5] Alonzi T, Middleton G, Wyatt S, Buchman V, Betz UA (2001). Role of STAT3 and PI 3-kinase/Akt in mediating the survival actions of cytokines on sensory neurons.. Mol Cell Neurosci.

[6] Gao Q, Wolfgang MJ, Neschen S, Morino K, Horvath TL (2004). Disruption of neural signal transducer and activator of transcription 3 causes obesity, diabetes, infertility, and thermal dysregulation.. Proc Natl Acad Sci U S A.

[7] Wu YY, Bradshaw RA (2000). Activation of the Stat3 signaling pathway is required for differentiation by interleukin-6 in PC12-E2 cells.. J Biol Chem.

[8] Zorina Y, Iyengar R, Bromberg KD (2009). Cannabinoid 1 Receptor and Interleukin-6 Receptor Together Induce Integration of Protein Kinase and Transcription Factor Signaling to Trigger Neurite Outgrowth.. Journal of Biological Chemistry.

[9] Shi CS (2004). Pyk2 Amplifies Epidermal Growth Factor and c-Src-induced Stat3 Activation.. Journal of Biological Chemistry.

[10] Quesnelle KM, Boehm AL, Grandis JR (2007). STAT-mediated EGFR signaling in cancer.. Journal of Cellular Biochemistry.

[11] Ng YP (2006). STAT3 as a Downstream Mediator of Trk Signaling and Functions.. Journal of Biological Chemistry.

[12] Reich NC, Liu L (2006). Tracking STAT nuclear traffic.. Nature Reviews Immunology.

[13] Decker T, Kovarik P (2000). Serine phosphorylation of STATs.. Oncogene.

[14] Yokogami K, Wakisaka S, Avruch J, Reeves SA (2000). Serine phosphorylation and maximal activation of STAT3 during CNTF signaling is mediated by the rapamycin target mTOR.. Curr Biol.

[15] Jain N, Zhang T, Kee WH, Li W, Cao X (1999). Protein kinase C delta associates with and phosphorylates Stat3 in an interleukin-6-dependent manner.. J Biol Chem.

[16] Chung J, Uchida E, Grammer TC, Blenis J (1997). STAT3 serine phosphorylation by ERK-dependent and -independent pathways negatively modulates its tyrosine phosphorylation.. Mol Cell Biol.

[17] Wegrzyn J, Potla R, Chwae YJ, Sepuri NB, Zhang Q (2009). Function of mitochondrial Stat3 in cellular respiration.. Science.

[18] Gough DJ, Corlett A, Schlessinger K, Wegrzyn J, Larner AC (2009). Mitochondrial STAT3 Supports Ras-Dependent Oncogenic Transformation.. Science.

[19] Vaudry D, Stork PJ, Lazarovici P, Eiden LE (2002). Signaling pathways for PC12 cell differentiation: making the right connections.. Science.

[20] Miranda C, Fumagalli T, Anania MC, Vizioli MG, Pagliardini S (2010). Role of STAT3 in In Vitro Transformation Triggered by TRK Oncogenes.. PLoS ONE.

[21] Sasagawa S, Ozaki Y, Fujita K, Kuroda S (2005). Prediction and validation of the distinct dynamics of transient and sustained ERK activation.. Nat Cell Biol.

[22] von Kriegsheim A, Baiocchi D, Birtwistle M, Sumpton D, Bienvenut W (2009). Cell fate decisions are specified by the dynamic ERK interactome.. Nat Cell Biol.

[23] Miller MW, Pitts FA (2000). Neurotrophin receptors in the somatosensory cortex of the mature rat: co-localization of p75, trk, isoforms and c-neu.. Brain Res.

[24] Johansson J, Formaggio E, Fumagalli G, Chiamulera C (2009). Choline up-regulates BDNF and down-regulates TrkB neurotrophin receptor in rat cortical cell culture.. NeuroReport.

[25] Wu YY, Bradshaw RA (1996). Induction of neurite outgrowth by interleukin-6 is accompanied by activation of Stat3 signaling pathway in a variant PC12 cell (E2) line.. J Biol Chem.

[26] Galli S, Jahn O, Hitt R, Hesse D, Opitz L (2009). A New Paradigm for MAPK: Structural Interactions of hERK1 with Mitochondria in HeLa Cells.. PLoS ONE.

[27] Lufei C, Ma J, Huang G, Zhang T, Novotny-Diermayr V (2003). GRIM-19, a death-regulatory gene product, suppresses Stat3 activity via functional interaction.. EMBO J.

[28] Verburg J, Hollenbeck PJ (2008). Mitochondrial membrane potential in axons increases with local nerve growth factor or semaphorin signaling.. J Neurosci.

[29] Ruthel G, Hollenbeck PJ (2003). Response of mitochondrial traffic to axon determination and differential branch growth.. J Neurosci.

[30] Hollenbeck PJ, Saxton WM (2005). The axonal transport of mitochondria.. J Cell Sci.

[31] Tso PH, Morris CJ, Yung LY, Ip NY, Wong YH (2009). Multiple Gi proteins participate in nerve growth factor-induced activation of c-Jun N-terminal kinases in PC12 cells.. Neurochem Res.

[32] Yung LY, Tso PH, Wu EHT, Yu JCH, Ip NY (2008). Nerve growth factor-induced stimulation of p38 mitogen-activated protein kinase in PC12 cells is partially mediated via Gi/o proteins.. Cellular Signalling.

[33] Rizzuto R, Simpson AW, Brini M, Pozzan T (1992). Rapid changes of mitochondrial Ca2+ revealed by specifically targeted recombinant aequorin.. Nature.

[34] Suzukawa K, Miura K, Mitsushita J, Resau J, Hirose K (2000). Nerve growth factor-induced neuronal differentiation requires generation of Rac1-regulated reactive oxygen species.. J Biol Chem.

[35] Cassano S, Agnese S, D'Amato V, Papale M, Garbi C (2010). Reactive oxygen species, Ki-Ras, and mitochondrial superoxide dismutase cooperate in nerve growth factor-induced differentiation of PC12 cells.. J Biol Chem.

[36] Lin W-F, Chen C-J, Chang Y-J, Chen S-L, Chiu I-M (2009). SH2B1β enhances fibroblast growth factor 1 (FGF1)-induced neurite outgrowth through MEK-ERK1/2-STAT3-Egr1 pathway.. Cellular Signalling.

[37] Liu RY, Snider WD (2001). Different signaling pathways mediate regenerative versus developmental sensory axon growth.. J Neurosci.

[38] He JC (2005). The G o/i-coupled Cannabinoid Receptor-mediated Neurite Outgrowth Involves Rap Regulation of Src and Stat3.. Journal of Biological Chemistry.

[39] Fricker A, Rios C, Devi L, Gomes I (2005). Serotonin receptor activation leads to neurite outgrowth and neuronal survival.. Molecular Brain Research.

[40] Poderoso C, Converso DP, Maloberti P, Duarte A, Neuman I (2008). A Mitochondrial Kinase Complex Is Essential to Mediate an ERK1/2-Dependent Phosphorylation of a Key Regulatory Protein in Steroid Biosynthesis.. PLoS ONE.

[41] Dagda RK, Zhu J, Kulich SM, Chu CT (2008). Mitochondrially localized ERK2 regulates mitophagy and autophagic cell stress: implications for Parkinson's disease.. Autophagy.

[42] Lee J, Sharma S, Kim J, Ferrante RJ, Ryu H (2008). Mitochondrial nuclear receptors and transcription factors: Who's minding the cell?. Journal of Neuroscience Research.

[43] McBride HM, Neuspiel M, Wasiak S (2006). Mitochondria: More Than Just a Powerhouse.. Current Biology.

[44] Salvi M, Brunati AM, Toninello A (2005). Tyrosine phosphorylation in mitochondria: A new frontier in mitochondrial signaling⋆.. Free Radical Biology and Medicine.

[45] Munnamalai V, Suter DM (2009). Reactive oxygen species regulate F-actin dynamics in neuronal growth cones and neurite outgrowth.. Journal of Neurochemistry.

[46] Min JY, Park MH, Park MK, Park KW, Lee NW (2006). Staurosporin induces neurite outgrowth through ROS generation in HN33 hippocampal cell lines.. Journal of Neural Transmission.

[47] Kotakenara E, Saida K (2007). Characterization of CoCl2-induced reactive oxygen species (ROS): Inductions of neurite outgrowth and endothelin-2/vasoactive intestinal contractor in PC12 cells by CoCl2 are ROS dependent, but those by MnCl2 are not.. Neuroscience Letters.

[48] Gopalakrishna R, Gundimeda U, Schiffman JE, McNeill TH (2008). A direct redox regulation of protein kinase C isoenzymes mediates oxidant-induced neuritogenesis in PC12 cells.. J Biol Chem.

[49] Wu H, Ichikawa S, Tani C, Zhu B, Tada M (2009). Docosahexaenoic acid induces ERK1/2 activation and neuritogenesis via intracellular reactive oxygen species production in human neuroblastoma SH-SY5Y cells.. Biochimica et Biophysica Acta (BBA) - Molecular and Cell Biology of Lipids.

[50] Kuroki M, O'Flaherty JT (1999). Extracellular signal-regulated protein kinase (ERK)-dependent and ERK-independent pathways target STAT3 on serine-727 in human neutrophils stimulated by chemotactic factors and cytokines.. Biochem J.

[51] Lim CP, Cao X (1999). Serine phosphorylation and negative regulation of Stat3 by JNK.. J Biol Chem.

[52] Brodie C, Bogi K, Acs P, Lazarovici P, Petrovics G (1999). Protein kinase C-epsilon plays a role in neurite outgrowth in response to epidermal growth factor and nerve growth factor in PC12 cells.. Cell Growth Differ.

